# The feasibility and effectiveness of thoracoscopic transdiaphragmatic approach for lung biopsy in rabbits. A randomized study ^1^

**DOI:** 10.1590/s0102-865020200050000001

**Published:** 2020-07-03

**Authors:** Monica Carolina Nery Wittmaack, Felipe Farias Pereira da Câmara Barros, Paloma do Espírito Santo Silva, Andresa Matsui, Guilherme Sembenelli, Renata Sitta Gomes Mariano, Pedro Paulo Maia Teixeira, Paola Castro Moraes

**Affiliations:** I MSc, Fellow PhD degree, Postgraduate Program in Science, Department of Veterinary Surgery, School of Agrarian Sciences and Veterinary Medicine (FCAV), Universidade Estadual Paulista (UNESP), Jaboticabal-SP, Brazil. Conception and design of the study; acquisition, analysis and interpretation of data; technical procedures.; II PhD, Associate Professor, Universidade Federal Rural do Rio de Janeiro (UFRRJ), Seropedica-RJ, Brazil. Acquisition of data, technical procedures.; III PhD, Veterinary Anesthesia Division, Department of Veterinary Surgery, FCAV, UNESP, Jaboticabal-SP, Brazil. Acquisition of data, technical procedures.; IV MSc, Department of Preventive Veterinary Medicine and Animal Reproduction, FCAV, UNESP, Jaboticabal-SP, Brazil. Histopathological examinations.; V MSc, Fellow PhD degree, Postgraduate Program in Science, Department of Veterinary Surgery, FCAV, UNESP, Jaboticabal-SP, Brazil. Acquisition of data, technical procedures.; VI PhD, Department of Preventive Veterinary Medicine and Animal Reproduction, FCAV, UNESP, Jaboticabal-SP, Brazil. Acquisition of data, technical procedures.; VII PhD, Full Professor, Veterinary Surgery Division, Universidade Federal do Pará (UFPA), Belem-PA, Brazil. Conception, design, intellectual and scientific content of the study.; VIII PhD, Full Professor, Department of Veterinary Surgery, FCAV, UNESP, Jaboticabal-SP, Brazil. Conception, intellectual and scientific content of the study; critical revision.

**Keywords:** Thoracic Surgery, Video-Assisted, Biopsy, Lung, Minimally Invasive Surgical Procedures, Rabbits

## Abstract

**Purpose:**

To assess the feasibility of thoracoscopic transdiaphragmatic approach for biopsy of all lung lobes and to determine the optimal intercostal space (ICS) for biopsy of each lung lobe.

**Methods:**

Ten rabbits were positioned in dorsal recumbency. Total thoracoscopy lung biopsy was made combined transdiaphragmatic approach and right ICS approaches. A camera port was made in the transdiaphragmatic approach and the instrument port was made of ICS 7 and ICS 9. A pre tied loop ligature was placed to performed a caudal lung lobe biopsy and to simulate biopsies of the others lung lobes.

**Results:**

Biopsy of the cranial aspect of the right caudal lung lobe was performed at ICS 9. Simulated biopsy of the accessory lung lobe was performed at ICS 9. Simulated lung biopsy of the right cranial and middle lung lobes was performed at ICS 7. The caudal and dorsal aspect of the right caudal lung lobe was not visualized by telescope at transdiaphragmatic approach, and biopsy was not performed.

**Conclusions:**

Thoracoscopic transdiaphragmatic approach for lung lobes biopsies was a feasible technique, except for the caudal aspect of the right caudal lung lobe. An ideal intercostal port for biopsy of each right lung lobe was determined.

## Introduction

Thoracoscopy is less invasive than open procedures. Indeed, many prospective, randomized studies conﬁrmed that thoracoscopy reduces postoperative pain, minimizes pulmonary dysfunction and shortens hospital stay^1 , 2^ . Recently, there has been an increasing enthusiasm for the thoracoscopic transdiaphragmatic approach. A transdiaphargmatic approach offers multiple benefits, including a reduction in the signs of pain attributable to spreading of the ribs, not producing lesion in the intercostal nerves, and preventing intercostal muscle tearing. Additionally, decrease intercostal approaches will reduce postoperative pain^3 - 5^ .

A thoracoscopic lung biopsy is considered to be the gold standard and is often suggested to clarify the diagnosis of a wide variety of lung diseases^6^ . The type of approach used to assess the chest depends on the desired result. Previous studies have reported the feasibility of a transdiaphragmatic approach for the lung biopsies for multiple lung involvement^7 , 8^ . However, a randomized study that confirms the biopsy of all pulmonary lobes via transdiaphragmatic approach in small animals has not yet been performed.

A technique for thoracoscopic transdiaphragmatic surgery for lung lobe biospsy in dogs and cats has been described^9 , 10^ . In those reports, thoracoscopic transdiaphargmatic surgery was performed by creating a paraxiphoid transdiaphragmatic approach and two intercostal approaches. However, details of the technique and an ideal ICS approach for lung biopsy of each lung lobe have not been described in small thoracic cavities. Thoracoscopic procedures in small patients are challenging^11^ ; the present study chose rabbits experimental models to reproduce the small size of the thoracic cavity, the smaller work area and the use of smaller instruments.

This experiment attempts to test the feasibility of thoracoscopic transdiaphragmatic approach for biopsy of all lung lobes in small thoracic cavity and to determine the optimal intercostal space (ICS) for biopsy of each lung lobe.

## Methods

The present study was approved by the Animal Use Ethics Committee (CEUA) of the School of Agrarian and Veterinary Sciences (FCAV) – UNESP – Campus Jaboticabal, and was carried out in accordance to the animal experimentation guidelines of CONCEA (protocol no. 12,900/15).

Ten adult male New Zealand white rabbits weighing between 3.0 and 4.0 kg were used. The animals came from a producer specialized in the species.

Prior to the thoracoscopy technique, all rabbits underwent radiographic evaluation of the thorax in the laterolateral and ventrodorsal projections. The radiographic images were used to verify the absence of possible lung disorders.

### Anaesthetic protocol

Initially, in order to perform the procedure, the rabbits received intramuscular pre-anesthetic medication composed of morphine (Dimorph®) (1 mg/kg) and acepromazine (PromAce®) (0.05 mg/kg).Anesthetic induction was performed with isoflurane (Isoflurano, Instituto Bioquímico), using an air-sealed face mask. After the animals were anesthetized, 10% spray lidocaine (Xylestesin®) was instilled into the oral cavity and, after dorsiflexion of the neck, orotracheal intubation was performed. Intubation was confirmed using a capnograph. The animals were maintained under spontaneous ventilation until the thorax was opened, after which assisted ventilation was initiated. Ventilation assistance was adjusted according to pulse oximetry (oxygen saturation) and capnography (end-tidal carbon dioxide [CO_2_] measurement). In this study, thoracic cavity insufflation was not performed.

### Surgical technique

The rabbits were positioned in dorsal recumbency, and aseptic preparation of the whole ventral and lateral thorax was made. This study standardized the right hemithorax for procedure. The first portal was inserted in the right intercostal space. A 0.5cm small incision was made through the skin and parallel to the cranial surface of the ribs. A Halsted hemostatic forceps was inserted for intercostal musculature divulsion and pleura perforation, with consequent induction of pneumothorax, in order to reduce the occurrence of iatrogenic lesions to the pulmonary parenchyma in the moment of insertion of the trocar. A 5-mm thoracoscopic cannula was inserted dorsal to the costochondral junction at ICS 9. Once the obturator was removed, a 4-mm 0° rigid endoscope was inserted into the thorax for initial evaluation of the thoracic cavity. The endoscope was positioned to evaluate all right lung lobes and visualize the diaphragm region for video-assisted insertion of the second trocar, by the transdiaphragmatic paraxiphoid approach.

Subsequently, a cutaneous incision was made with approximately 0.5cm of extension, longitudinally to the ribs, next to the xiphoid process (paraxiphoid). Allis hemostatic forceps was grabbing and elevate cutaneous, subcutaneous and muscular tissue before cutaneous incision by blade, in order to reduce the occurrence of iatrogenic lesions to the pulmonary parenchyma in the moment of insertion of the trocar. A blunt trocar-cannula was inserted directed cranially, and slightly laterally and ventrally, to avoid the mediastinum. The site of transdiaphragmatic approach was carefully chosen on the muscular pars of the right diaphragmatic dome. After the video-assisted perforation of the diaphragm, the endoscope was removed from the intercostal approach and positioned through the transdiaphragmatic approach. The Babcock (atraumatic) grasping forceps for lung clamping was inserted through the portal at ICS 9.

**Figure 1:**
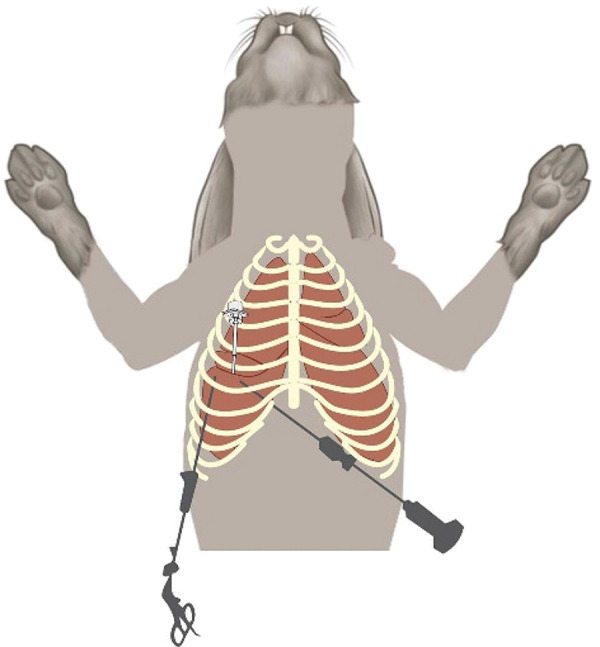
- Schematic representation of the thoracoscopic transdiaphragmatic approach in a rabbit. The first port is placed in a paraxiphoid position and instrument ports are placed in the intercostal spaces.

The third portal was made for pre tied loop ligature. The third trocar with 3mm of diameter was inserted ventral to the costochondral junction perpendicularly to the thoracic wall. The pre tied loop ligature was placed at an angle into to allow application of the suture as near as possible to the lung lobe, using the transillumination of the skin as a guide to facilitate biopsy. In this study, the third portal for pre tied loop ligature was made in the ICS 7 ( Fig. 1 ).

### Lung biopsy

**Figure 2:**
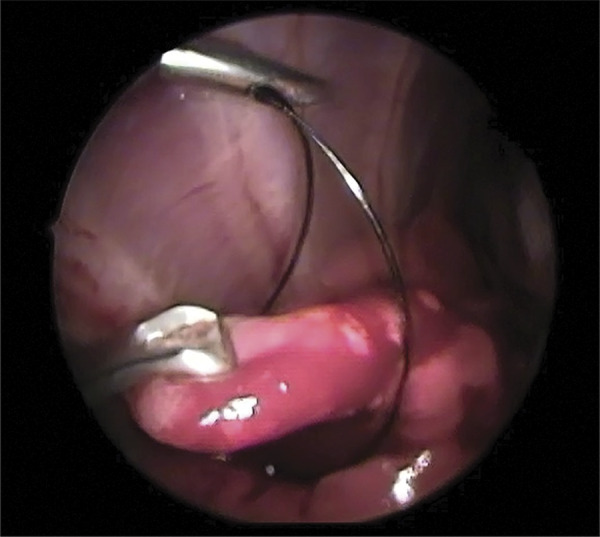
- Thoracoscopic lung biopsy using pre tied loop ligature in a rabbit. The periphery of a caudal lung lobe is encircled with a pretied loop ligature and grasped with Babcock forceps for sampling.

The telescope was moved caudally so that each lung lobe could be explored. The cranial aspect of right caudal lung lobe was evaluated for telescope via transdiafragmatic approach. Peripheral right caudal lung biopsy was done by applying a pre tied loop ligature. A Babcock forceps pass through the loop and elevate the tip of lung, which is ligated by tightening the loop ( Fig. 2 ). The pre tied loop ligature was removed and endoscopic scissors was inserted to transect the tip of lung and severed the suture end. The caudal lobe lung was evaluated for hemorrhage and air leakage after sampling. Lung biopsy of the caudal aspect of right caudal lung lobe was not possible via transdiphragmatic approach.

Procedure was repeated at ICS 9 on the right side for simulated lung biopsy of the accessory lung lobe. The accessory lung lobe was grabbing by Babccok forceps, passed through the loop to simulate lung biopsy. In order to simulated lung biopsy of the right cranial and right middle lung lobes, a 5mm port at ICS 9 was removed and reinserted at ICS 7 on the right side dorsal to the costochondral junction and lung biopsy was simulated using pre tied loop ligature.

Thus, each of the 10 rabbits underwent to lung biopsy via thoracoscopic transdiaphragmatic approach and intercostal approach, and better ICS for each lung lobe biopsy was tested (ICS 7 and ICS 9 on the right hemithorax). And right cranial, right middle and accessory lung lobe biopsies were simulated; an actual lung lobe biopsy was performed only in right caudal lung lobe. A pre tied loop ligature was used for simulated lung biopsies in the study reported here. No ligature was placed for any of the simulated biopsies. An ICS was selected based on the subjectively ease to perform lung biopsy, avoided iatrogenic damage to vessels and pulmonary parenchyma, and did not require specialized equipment.

### Closure and postoperative care

After the procedures, the portals were removed. The intercostal musculature was sutured with 3 metric polyglactin 910 (Vicryl; Ethicon) using interrupted absorbable sutures and the skin was sutured with 3 metric nylon (Ethilon; Ethicon) using simple interrupted sutures. Prior to the last suture, negative thoracic pressure was reinstated using recruitment maneuver. After the last skin suture, thoracocentesis was performed on the 8^th^ intercostal space with 23 gauge butterfly needle to ensure no intrathoracic air or liquid were present.

The animals were medicated with subcutaneous tramadol hydrochloride (Tramal®) (4 mg/kg) every 8 hours for five days, subcutaneous meloxicam (Maxicam® 0.2%) (0.1 mg/kg) every 24 hours for two days, and subcutaneous enrofloxacin (Chemitryl® 2.5%) (5 mg/kg) every 12 hours for seven days.

### Tissue collection and fixation

Samples were measured, identified, kept in a 10% buffered formalin solution and then submitted for histological analysis. All samples were evaluated by the same pathologist through the Hematoxylin-Eosin staining technique.

### Postoperative assessment

The animals underwent thoracic radiography in three projections in the immediate post-operative period and on the second and fourth days after the surgery. Thorax radiographs taken before and after the surgeries were compared to verify the absence of pneumothorax or hemothorax. Respiratory parameters were assessed daily for 15 days. In addition, possible changes such as the presence of subcutaneous emphysema, seroma, local infection and dehiscence of stitches were observed. After fifteen days from the procedure postmortem examination was made.

## Results

### Surgical results

Total thoracoscopic lung biopsy was made combining transdiaphragmatic approach and intercostal approach. An optimal ICS for biopsy of each right lung lobe was determined. Biopsy of the cranial aspect of caudal lung lobe was performed at ICS 9. The caudal and dorsal aspect of the caudal lung lobe was not evaluated under direct visualization by telescope at transdiaphragmatic approach, and biopsy was not performed. Simulated biopsy of the accessory lung lobe was performed at ICS 9. Simulated lung biopsy of the right cranial and middle lung lobes was performed at ICS 7.

The right intercostal approach and transdiaphragmatic paraxiphoid approach were made without major difficulties. The option to insert the second portal through the transdiaphragmatic paraxiphoid approach under direct visualization by endoscope was safe, without the occurrence of iatrogenic lesion in any of the 10 animals. No conversion was required for thoracotomy and all remained alive during the analyzed period.

### Necropsy

**Figure 3:**
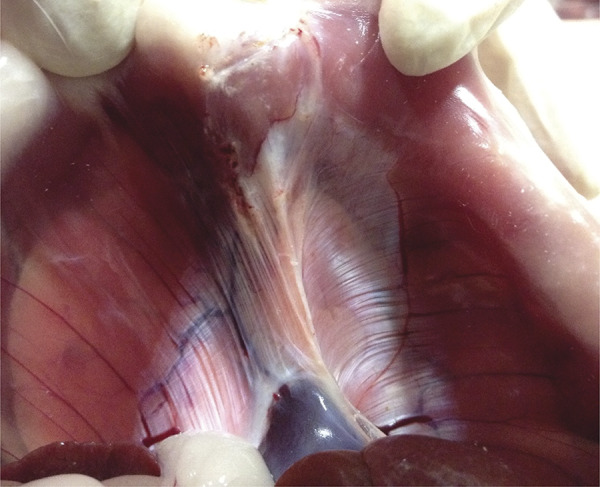
Postmortem examination of diaphragm. Note the small scar in the diaphragm pars muscularis.

The postmortem examination 15 days after surgery revealed complete healing of the diaphragmatic incision ( Fig. 3 ). At necropsy, the lung biopsies were completely healed. There were no signs of infection in all animals. Three animals had adhesion to the diaphragm; however, all of them had completed healing. Other animals had no adhesion in both thoracic and peritoneal cavity.

### Histologic evaluation

The lung biopsy samples were measured in 3 dimensions, and the mean volume of the biopsy specimens was 35 cm^3^ (range, 5 to 68 cm^3^ ). According to the histopathology report, the samples obtained were considered satisfactory and allowed the anatomic structures of the lung (bronchi, bronchioles, and alveoli) to be visualized.

### Clinical and radiographic findings

In the postoperative clinical evaluation of all animals, no respiratory distress, tachypnea or other clinical signs related to the respiratory tract were found during the study. The rabbits did not present subcutaneous emphysema and seroma. No complications of the surgical wound were found, such as infection and dehiscence.

Postoperatively, results of thoracic radiography were normal in all animals, and none presented pneumothorax. Pleural effusion was also not identified after procedures.

## Discussion

A trandiaphragmatic approach has potential advantages such as preventing injuries to intercostal nerves and vessels due to the bypass of the intercostal space during thoracoscopic surgery^4 , 12^ . Commonly, a transdiaphragmatic approach was indicated as appropriated approach for a lung biopsy because of diffuse lung disease. This approach allows examination of both sides of the thoracic cavity and biopsy of the lungs on the right and left sides^7 , 8^ . However, a recent study did not recommend transdiaphragmatic approach for evaluation of the dorsal aspect of the chest^11^ . In the present study, biopsy of all right lungs lobe at transdiaphragmatic approach was not possible. Caudal and dorsal aspect of the caudal lung lobe was not evaluated under direct visualization by telescope at transdiaphragmatic approach, even moving the telescope caudally and dorsally.

In the current study, the initial approach to the thorax was performed through intercostal approach. Studies warn about the technical challenges during the insertion of the first portal with no visualization of the optics. The transdiaphragmatic entrance of the first trocar in the thoracic cavity can lead to lung lesions and lacerations^12 , 13^ . Building on these ﬁndings, the initial approach to the thorax was performed in order to provide greater safety, reducing the risks of iatrogenesis to the intrathoracic structures that could be caused by blind transdiaphragmatic paraxiphoid approach. Furthermore, the first portal was inserted by minithoracotomy (5mm) and provided insertion of trocars with little resistance, adequate pneumothorax and avoided possible iatrogenic lesions on the lung, as described in several other studies^10 , 14^ . This maneuver also allowed to the extent of thoracic cavity exploration. All lungs lobes were not evaluated under direct visualization by telescope at transdiaphragmatic approach, only when telescope was at intercostal approach^9^ .

Another consideration was that a transdifragmatic approach usually requires division of the mediastinum for entry and examination of the contralateral hemithorax. For this, an instrument port placed at intercostal space in the ipsilateral hemithorax is necessary to divide the ventral mediastinum, after which the contralateral hemithorax can be examined^10^ . Therefore, the thoracoscopic transdiaphragmatic approach often requires an intercostal portal placed before the complete exploration of the two hemithoraxes. In this study, the option of first port in ICS to evaluated right hemithorax permits to convert transdiafragmatic approach to traditional transthoracic approach if lesion is located in caudal and dorsal aspect of the caudal lung lobe.

Regarding the size and quality of the material collected in the present study, the samples obtained were considered satisfactory, and it was possible to assume the same diagnosis for the pulmonary lobes in which the biopsies were simulated. The results of this study corroborate previous studies that performed lung lobe biopsy in dogs and cats^15 - 17^ .

Previous studies highlight the diagnostic character of the thoracoscopic procedures as one of the advantages that this access allows^18 , 19^ . However, for greater sensitivity and specificity, the optimal pre determination of thoracoscopic approach is recommended^20^ . Simulated lung biopsy of the right cranial and right middle lung lobes performed at ICS 9, was subjectively more difficult compared with lung biopsy performed at ICS 7. Short distance between the Babcock forceps and the right cranial and middle lung lobes at ICS 7 improved the safety of the technique. In all rabbits the ICS portal had to be reinserted into ICS 7 to simulated cranial and middle lung biopsy. The instrument portal was placed 1 to 3 intercostal spaces caudal to the lung lobe that will be biopsied. This was accomplished to account for the space between the lung and the relatively fixed small thoracic space.

In the present investigation, thoracoscopic procedure without the additional insufflation of CO_2_, was the chosen option. A recent review emphasizes the thoracoscopic procedures are done within the rigid thorax and insufflation is not required^10 , 11^ . The intrathoracic inflation with carbon dioxide in thoracoscopic procedures is not essential and promotes small distension of the chest cavity. In addition, it can cause an increase in blood pressure and compromise circulatory dynamics^11 , 21^ . In addition, one-lung ventilation and endobronchial blockade are not standard for thoracoscopy. One-lung ventilation may be necessary for pneumolobectomy, but is not usually used to decrease pulmonary trauma, because of its consequences with ventilation. Also, when one-lung ventilation is essential, it requires intensive monitoring and specialized equipment^20^ . Simply creating a pneumothorax and altering the means of ventilation usually creates enough working space for thoracoscopy^11 , 22 , 23^ .

Limitations of this study include the thoracic conformation differences between rabbits, canines and felines, despite the similar body weight. Another limitation included that the data in this study were only about right hemithorax. Additional constraint includes only right caudal lung lobe was biopsied and objective lung biopsy for others lobes were not made, only simulation; thus, further research is warranted. In addition, the present study includes the use of experimental animals that did not have pulmonary pathologies, which may not accurately mimic clinical situations. Finally, the data provided in the experimental study reported here suggest the optimal location for biopsy of each lung lobe without carbon dioxide insufflation of the thoracic cavity, in order to prevent cardiopulmonary damage. However, results may vary when the technique is applied with different insufflation pressures.

## Conclusions

Results for the study reported here may provide surgeons with information on the optimal ICS approach to use when performing lung biopsy via transdiaphragmatic approach in patients less than 5kg. Further evaluation of a thoracoscopic transdiaphragmatic approach for biopsy of right caudal lung lobe is required in small dogs and cats.

## Footnotes

1 Research performed at Endoscopy Laboratory, Department of Veterinary Clinic and Surgery, Veterinary Hospital, Universidade Estadual Paulista (UNESP), Jaboticabal-SP, Brazil.
